# A novel method to recover inclusion body protein from recombinant *E. coli* fed-batch processes based on phage *Φ*X174-derived lysis protein E

**DOI:** 10.1007/s00253-017-8281-x

**Published:** 2017-04-20

**Authors:** Daniela Ehgartner, Patrick Sagmeister, Timo Langemann, Andrea Meitz, Werner Lubitz, Christoph Herwig

**Affiliations:** 1 Institute for Chemical, Environmental and Biological Engineering, TU Wien, Gumpendorferstrasse 1a/166, 1060 Vienna, Austria; 2 CD Laboratory on Mechanistic and Physiological Methods for Improved Bioprocesses, TU Wien, Gumpendorferstrasse 1a/166, 1060 Vienna, Austria; 3 Exputec GmbH, Pfeilgasse 32/20, 1080 Vienna, Austria; 4 RCPE–Research Center of Pharmaceutical Engineering GmbH, Inffeldgasse 13, 8010 Graz, Austria; 5 BIRD-C GmbH, Dr.-Bohr-Gasse 2-8, 1030 Vienna, Austria

**Keywords:** Bioprocess technology, Recombinant protein release, *Escherichia coli*, pBAD expression system, Mixed feed bioprocesses, Bacterial Ghost

## Abstract

Production of recombinant proteins as inclusion bodies is an important strategy in the production of technical enzymes and biopharmaceutical products. So far, protein from inclusion bodies has been recovered from the cell factory through mechanical or chemical disruption methods, requiring additional cost-intensive unit operations. We describe a novel method that is using a bacteriophage-derived lysis protein to directly recover inclusion body protein from *Escherichia coli* from high cell density fermentation process: The recombinant inclusion body product is expressed by using a mixed feed fed-batch process which allows expression tuning via adjusting the specific uptake rate of the inducing substrate. Then, bacteriophage *Φ*X174-derived lysis protein E is expressed to induce cell lysis. Inclusion bodies in empty cell envelopes are harvested via centrifugation of the fermentation broth. A subsequent solubilization step reveals the recombinant protein. The process was investigated by analyzing the impact of fermentation conditions on protein E-mediated cell lysis as well as cell lysis kinetics. Optimal cell lysis efficiencies of 99% were obtained with inclusion body titers of >2.0 g/l at specific growth rates higher 0.12 h^−1^ and inducer uptake rates below 0.125 g/(g × h). Protein E-mediated cell disruption showed a first-order kinetics with a kinetic constant of −0.8 ± 0.3 h^−1^. This alternative inclusion body protein isolation technique was compared to the one via high-pressure homogenization. SDS gel analysis showed 10% less protein impurities when cells had been disrupted via high-pressure homogenization, than when empty cell envelopes including inclusion bodies were investigated. Within this contribution, an innovative technology, tuning recombinant protein production and substituting cost-intensive mechanical cell disruption, is presented. We anticipate that the presented method will simplify and reduce the production costs of inclusion body processes to produce technical enzymes and biopharmaceutical products.

**Electronic supplementary material:**

The online version of this article (doi:10.1007/s00253-017-8281-x) contains supplementary material, which is available to authorized users.

## Introduction

The gram-negative bacterium *Escherichia coli* is the primary microbial cell factory (Andersen and Krummen 2002; Carneiro et al. 2012; Choi et al. 2006) producing 39% of all recombinant proteins on the market (Demain and Vaishnav 2009). Recombinant proteins in *E. coli* can either be produced in soluble form or as insoluble protein aggregates, referred to as inclusion bodies. Soluble proteins are usually active, while protein in inclusion bodies is often reported to be misfolded and hence inactive (Sorensen and Mortensen 2005). More recent investigations showed that a big part of proteins in inclusion bodies is functional and can thereby be used for multiple applications beforehand without being solubilized and refolded (Villaverde et al. 2015). The production of inclusion bodies is preferred in many cases since inclusion bodies can accumulate in the cytoplasm in higher quantities than soluble proteins, and the recombinant product can be isolated in a highly concentrated and purified state (Baneyx and Mujacic 2004; Choi et al. 2006).

*Escherichia coli* does not possess a native outer membrane transporter (Francetic et al. 2000) which makes cell rupture necessary to isolate recombinant products. Classical cell rupture methods used to release the protein (soluble or in the form of inclusion body aggregates) from the cytoplasm are the following: detergent treatment, cell rupture by lysozyme, sonication, and freeze-thaw (Bird et al. 2004), as well as high-pressure homogenization, bead milling, and thermolysis (Ren et al. 2007).

The phage ϕX174-derived lysis protein E is able to build a transmembrane tunnel structure by fusing inner and outer membranes in Gram-negative bacteria (Witte et al. 1990a, b). Border values of the tunnel are between 40 and 200 nm. Due to the osmotic pressure difference between the cytoplasm and the bacterial environment, the cell content is discharged (Witte et al. 1990a). Empty envelopes of Gram-negative bacteria, emerged by controlled expression of the cloned lysis gene E, are referred to as Bacterial Ghosts (BGs) (Lubitz et al. 1999). BGs find multiple biotechnological applications as microbioreactors, carriers of DNA vaccines (Langemann et al. 2010), or whole-cell biocatalysts (Sührer et al. 2015). The discharge of cytoplasmatic content due to protein E-mediated lysis promises an application of phage ϕX174-derived lysis protein E for recombinant protein release. The capability of cells to perform E-mediated lysis (E-lysis) is, however, dependent on the physiological state of the cell and its ability to divide (Blasi et al. 1985; Lubitz et al. 1984). The latter is negatively influenced by the metabolic load during recombination protein production.

The so-called metabolic load is an alteration in the carbon metabolism of the host cells, induced by recombinant protein production (Heyland et al. 2011). This stress response, that occurs due to recombinant protein production, is linked to the rates of transcription but can also be intensified by specific properties of the protein, e.g., misfolding (Hoffmann and Rinas 2004). A high metabolic burden can furthermore decrease the viability of cell population (Andersson et al. 1996; Glick 1995) and promote the formation of subpopulations of viable but non-dividing cells (Andersson et al. 1996). Using bacteriophage ϕX174-derived lysis protein E in this context is highly critical as the applicability of this technology requires the cells to be in a viable and culturable state (Halfmann and Lubitz 1986). Reducing the metabolic load on the cell factory is hence a potentially vital step for this technology to work.

Mixed feed processes are one possibility to decrease the metabolic load during recombinant protein expression. These processes are reported to enable tuning of recombinant protein expression rate. Mixed feed bioprocesses are characterized by the controlled feeding of two or more different carbon sources in a defined ratio. These systems are applied in the means that the first carbon source is linked to cell growth and cell energy, whereas, the second carbon source is used to regulate recombinant protein production (Sagmeister et al. 2013a). Mixed feed systems were shown to increase productivity of heterologous products (Anjou and Daugulis 2000; Jungo et al. 2007; Wurm et al. 2016).

The pBAD mixed feed system is based on the simultaneous feeding and metabolizing of D-glucose as primary and L-arabinose as secondary carbon source. Thereby, L-arabinose acts as inducer of recombinant gene expression (Sagmeister et al. 2013a, 2014). Although L-arabinose suffers from catabolite repression in case D-glucose is present in excess (Siegele and Hu 1997), *E. coli* is able to metabolize L-arabinose and D-glucose simultaneously in a wide range of specific uptake rates (Sagmeister et al. 2013a). The level of expression can be modulated by the specific uptake rate of L-arabinose. Sagmeister et al. (2014) applied the pBAD mixed feed expression system to demonstrate tuning of the transcription rate on a cellular level via adjusting the uptake rate of L-arabinose. In order to do so, green fluorescent protein (GFP) was used as a model product (Sagmeister et al. 2014).

We aim to present a generic method for the recovery of recombinant protein from inclusion bodies from high-density fed-batch processes that is based on the controlled expression of bacteriophage ϕX174-derived lysis protein E. Thereby, inclusion body protein isolation should be simplified and present an alternative to common mechanic or chemical cell rupture.

To enable protein E-mediated cell lysis in high-level recombinant processes, we aim at tuning recombinant protein expression using the pBAD mixed feed system to reduce the metabolic load on the cell factory. As a response, the basic E-lysis kinetics, product purity, and titer are investigated. This allows to scientifically design industrial inclusion body processes by using the presented technology.

## Materials and methods

### Bioprocess

#### The strain

An *E. coli* C41 (OverExpress™ C41 (DE3)): F– ompT hsdSB (rB−, mB−) gal dcm (DE3) strain (Lucigen, Middleton, Wisconsin, USA; provided by BIRD-C, Vienna, Austria) was used. It contained two plasmids coding for the recombinant human bone morphogenetic protein 2 (rhBMP-2, NCBI gene ID: 650) as well as the phage protein E.

The genes for recombinant protein production were on the plasmid pBK-BMP, which originates from pBAD24 (Guzman et al. 1995; provided by BIRD-C, Vienna, Austria) and is shown in Fig. S1 in the supplementary material. RhBMP-2 production was induced by L-arabinose, which additionally served as the secondary C-source. The gene E cassette was on pGLysivb (provided by BIRD-C, Vienna, Austria). This plasmid carried a part from a gentamycin resistance cassette, namely the lysis gene E controlled by a temperature-inducible λ_pR_, _mut_-cI857 P/O-system (Jechlinger et al. 2005; Langemann et al. 2016). The expression of lysis gene E and thus the start of E-lysis was induced by increasing the cultivation temperature to 42 °C.

#### Media

Media were used as described in DeLisa et al. (1999). The batch medium was complemented with 20 mg/l gentamycin and 20 mg/l kanamycin. For the fed-batch phase, the feed containing 400 g/l D-glucose was the only substrate (described in DeLisa et al. (1999)). During the induction phase, D-glucose and L-arabinose were combined in the feed. The total sugar concentration was 400 g/l. The amount of each of these two sugars in the feed was calculated in order to achieve the ratio of a specific substrate uptake rate (*q*_s_) of D-glucose and of L-arabinose. As *q*_s_ D-glucose and *q*_s_ L-arabinose differed between cultivations, so did the ratio of the sugars in the medium.

#### Upstream process

Multiple fermentations were conducted in a Techfors-S bioreactor (Infors, Bottmingen, Switzerland) of 10-l maximum working volume with a bioreactor setup and instrumentation as described elsewhere (Ehgartner et al. 2015).

The culture was grown at a constant of pH 7.2 and 35 °C. Dissolved oxygen was regulated at a level higher than 30% by adding oxygen to the inflowing air. The total gas flow into the bioreactor was constant at 1.5 vvm. The cultivation included a batch, an uninduced fed-batch, an L-arabinose pulse, and an induced fed-batch phase (described more in detail in Ehgartner et al. (2015)). Furthermore, an E-lysis phase was done after the induced fed-batch had been finished, whereas, temperature was shifted to 42 °C for E-lysis induction.

Parallel to starting the fed-batch, a first-principle soft sensor based on elemental balancing was initialized. To do this, the biomass concentration was estimated via OD correlation as a starting value, a procedure more thoroughly described elsewhere (Sagmeister et al. 2014). The uninduced fed-batch was conducted with a feed-forward strategy at a specific growth rate of 0.2 h^−1^ until an estimated biomass concentration of 15 g/l was reached. In this phase of the process, D-glucose functioned as the sole carbon source. After the uninduced fed-batch, an L-arabinose pulse was performed to a final concentration of 2.5 g/l to adapt the cells. During this period, no additional sugar was fed.

**Fig. 1:**
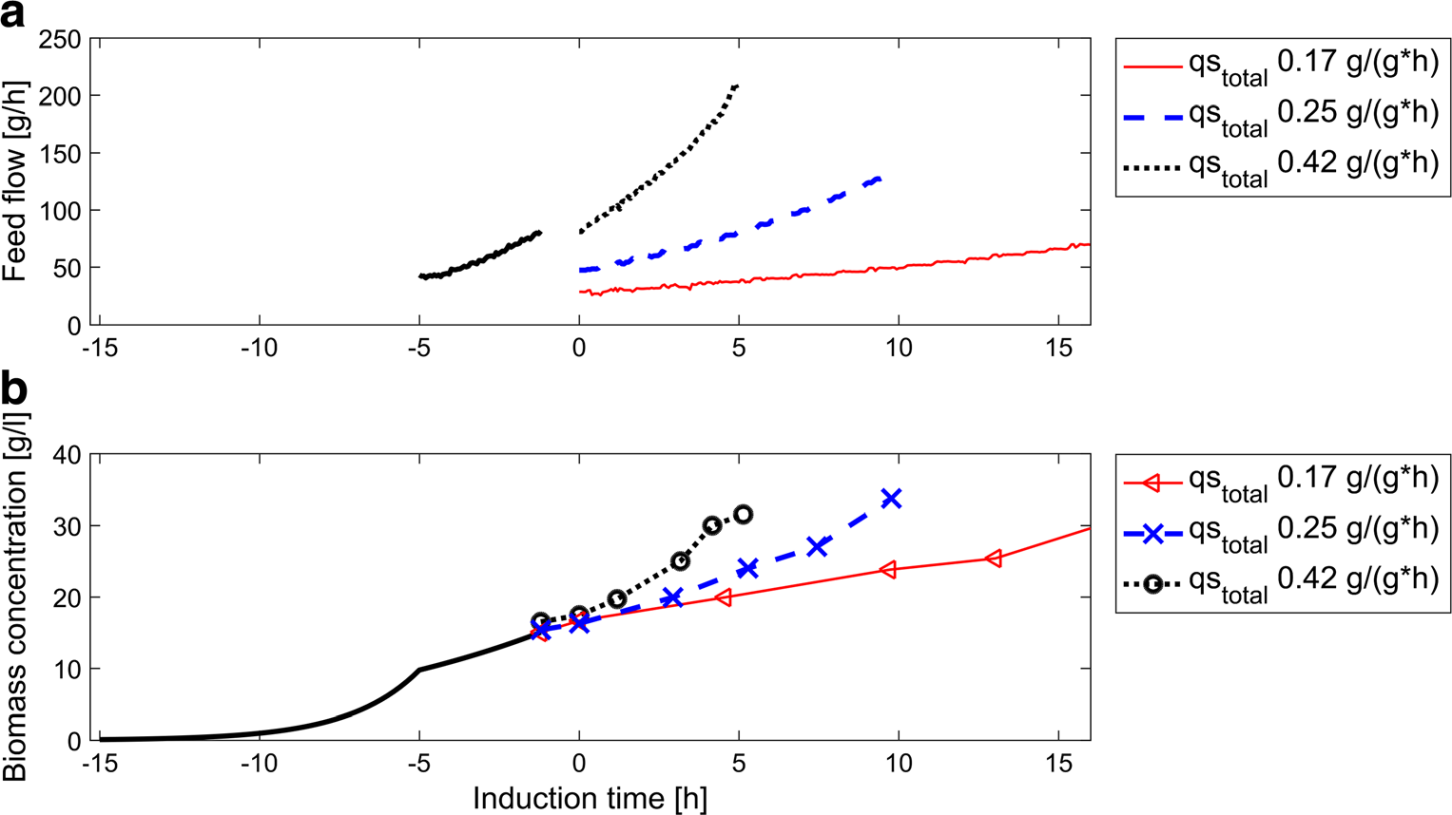
An overview **a** over the feed flow and **b** biomass concentrations during uninduced and induced fed-batch for three cultivations with different total *q*_s_. The start of induction phase is indicated as time point *0*. The gap between uninduced fed-batch and induction phase represents the L-arabinose pulse. In processes with higher *q*_s_, the induction phase was shorter as a biomass concentration of 35 g/l was reached earlier. During uninduced fed-batch, feeding rates were the same for all cultivations, which is shown by the *black line*

During the induction phase, a mixed feed (D-glucose and L-arabinose) was used. The specific substrate uptake rates of D-glucose and L-arabinose varied between cultivations based on a design of experiments (DoE) to investigate the influence on product titer, on product purity, and on E-lysis. To achieve the desired set points of *q*_s_ L-arabinose and *q*_s_ D-glucose, the ratio of D-glucose and L-arabinose in the feed was prepared respectively. The total specific substrate uptake rate was controlled by a soft-sensor-assisted closed loop control strategy and was set by the feeding rate. Figure 1 exemplifies how the feeding rate and biomass concentration in the induction phase differed in three runs with varying total *q*_s_. The duration of the induction phase varied depending on the total specific substrate uptake rate since all fermentations were grown to the same biomass concentration.

As soon as the estimated biomass concentration in the induction phase approached a value of 35 g/l, the feed was changed back to D-glucose feed, which had been previously used for the non-induced fed-batch. The reactor temperature was shifted to 42 °C to induce protein E formation and, consequently, protein E-mediated lysis of the cellular population. An annular type Permittivity probe (Aber Instruments, Abersystwyth, Great Britain) was used for biomass measurement during the E-lysis phase and, hence, was the basis for the control of the specific D-glucose uptake rate. Biomass estimation via Permittivity measurement was conducted as described in Meitz et al. (2016), with the difference that the calibration was done using an at-line determined biomass concentration via OD measurements. During the E-lysis phase, samples were taken every 30 min. The process was stopped after 180-min E-lysis phase.

The batch, the uninduced fed-batch, the L-arabinose pulse, and the E-lysis phase were the same for all cultivations. Only the induced fed-batch phase differed in *q*_s_ D-glucose and *q*_s_ L-arabinose as described in the following section.

#### Design of experiments

Recently, we reported induced state metabolic capabilities in respect to catabolite repression (maximum uptake of L-arabinose in the presence of uptake of D-glucose) for the pBAD mixed feed system (Sagmeister et al. 2013a). Based on these results, an orthogonal experimental central composite design, involving the specific D-glucose uptake rate as well as the specific L-arabinose uptake rate, was designed. Set points for specific substrate uptake rates for both D-glucose and L-arabinose were set to be 0.05, 0.125, and 0.2 g/(g × h). The total specific substrate uptake rate thus varied between 0.1 and 0.4 g/(g × h).

Due to primary results of the DoE cultivations, the DoE was slightly changed. In total, ten fermentations were conducted, including different combinations of these three *q*_s_ levels for both substrates. Additionally, the center point (fed-batch 6/7) and the high point *q*_s_ D-glucose/low *q*_s_ L-arabinose (fed-batch 1/2) were conducted in duplicate to evaluate the experimental replicate error. In fed-batch 2, the process was stopped after the induction phase. An overview of the cultivations and process conditions is given in Table 1.

**Table 1:** Overview over cultivations—process variables and outcomes

FB ID	*q*_s_ gluc (g/(g × h))	*q*_s_ ara (g/(g × h))	*q*_s_ total (g/(g × h))	*x* ara (%)	*μ* (1/h)	Titer (g/l)	*k* (−)	LE (%)	Purity hom. (%)	Purity lysed (%)
1	0.220	0.055	0.275	20.0	0.08	3.2	–	–	70	–
2	0.240	0.059	0.299	19.8	0.10	2.5	−.54	81.9	61	53
3	0.263	0.143	0.406	35.1	0.12	2.1	−1.25	99.0	50	47
4	0.202	0.222	0.423	52.3	0.16	1.5	−.52	90.2	55	41
5	0.119	0.051	0.179	28.4	0.05	4.0	−.74	75.5	58	55
6	0.127	0.127	0.254	50.0	0.09	3.5	−.47	61.7	61	54
7	0.131	0.141	0.272	51.9	0.10	3.8	−.7	47.4	57	60
8	0.135	0.227	0.362	62.7	0.12	2.5	−.92	75.6	49	43
9	0.080	0.087	0.168	52.1	0.05	6.1	−.97	39.3	71	59
10	0.049	0.152	0.200	75.8	0.07	5.3	−0.68	31.0	79	58

The cultivations are numbered with a fed-batch ID (FB ID). Values for the mean *q*_s_ D-glucose (*q*_s_ gluc), *q*_s_ L-arabinose (*q*_s_ ara), *q*_s_ total, and specific growth rate (*μ*) during the induction phase are given. Furthermore, the L-arabinose concentration in the mixed feed (*x* ara) is shown. The rhBMP-2 titer at the end of the induction phase (titer) represents the productivity in each process. Information about E-lysis can be taken from the maximal lysis efficiency (LE) and the lysis reaction coefficient *k*. The purity is presented for homogenized samples at the end of the induction phase (purity hom.) and an E-lysed non-homogenized sample at maximal E-lysis efficiency (purity lysed)

#### Process control using a first-principle soft-sensor

In this study, a previously reported soft-sensor control strategy designed for process development was applied (Sagmeister et al. 2013b). In short, an overdetermined equation system involving the carbon and degree of reduction balance was established for the estimation of metabolic rates (carbon dioxide formation rate, oxygen uptake rate, carbon substrate uptake rate). These reconciliation rates were used to estimate the unknown biomass formation rate. The algorithm for reconciliation was adapted from literature (van der Heijden et al. 1994). The biomass concentration was estimated by numerically integrating the estimated biomass formation rate. Volume calculation was based on the mass balance, whereas, the change of volume was calculated based on mass in-flows minus out-flows. For the control of the specific substrate uptake rate by the soft-sensor, the estimated biomass was used to calculate the appropriate flow rate (Eq. 1), which was regulated via a PI flow controller.

1 $$ {F}_0=\frac{{q_s}^{\ast }{c_x}^{\ast }{\uprho_{feed}}^{\ast }{V}_R}{c_s} $$

Equation 1: Calculation of the feed flow rate. The equation contains the specific substrate uptake rate (*q*_s_), the biomass concentration (*c*_*x*_), the density of the feed (*ρ*_feed_), the volume of the cultivation broth (*V*_R_), and the concentration of substrate in the feed (*c*_s_).

More details about the first-principle soft-sensor applied in this study are found in Sagmeister et al. (2013b) and Wechselberger et al. (2013).

### Analytics

#### Biomass dry cell weight

The dry cell weight concentration (DCW) determination was done in duplicate. A 2-ml sample was centrifuged at 4332×*g*, 10 min, and 4 °C. The supernatant was put on −20 °C for later investigation while the pellet was washed, centrifuged again, and then dried for 48 h at 105 °C. The DCW was determined gravimetrically.

#### Homogenization and cell rupture

A duplicate of 2-ml fermentation broth was centrifuged (4332×*g*, 10 min, 4 °C) and washed once. For homogenization, the pellets were resuspended to 20 ml in a buffer (50 mM Tris, 5 mM DTT, 1 mM EDTA, pH 8). All samples were suspended in the buffer, but only samples taken before the E-lysis phase were homogenized (Avestin EmulsiFlex©, Ottawa, Canada) in a continuous mode at 1500 bar for 3 passages.

#### Purity determination of rhBMP-2 via SDS gel analysis

For purity determination, homogenized samples and lysed samples were used. For better comparability, the samples taken after E-lysis were solved in homogenization buffer. One milliliter of each of these samples was centrifuged (16,600×*g*, 10 min, 4 °C). For homogenized samples, the resulting pellet and the supernatant were separately reduced on a heating block (5 min, 95 °C) in a buffer described by Laemmli (1970). The pellets of the lysed samples and the supernatant of the centrifugation for DCW determination were treated in the same way. Thirty microliters of each sample, as well as 10 μl standard (SeaBlue® Plus2 Pre-Stained; Invitrogen, Carlsbad, CA, USA), was loaded onto sodium dodecyl sulfate polyacrylamide gel electrophoresis (SDS) gels (8–16%; GE Healthcare, Chalfont St. Gilles, Great Britain).

After running the gels (160 V, 60 min), they were stained overnight in a sensitive Coomassie solution (0.02% (*w*/*v*) Coomassie Brilliant Blue G 250, 5% (*w*/*v*) aluminum sulfate-(14–18)-hydrate, 10% (*v*/*v*) ethanol, 2% (*v*/*v*) orthophosphoretic acid, distilled water). Stained gels were scanned and analyzed using the software ImageLab (Bio-Rad, Hercules, CA, USA). To investigate the purity of rhBMP-2, reference bands were marked and the intensity of the bands was then compared. The reference bands were selected in respect to their impact on downstream processing. Each sample was evaluated in triplicate on three different gels.

#### rhBMP-2 titer investigation via RP-HPLC

For rhBMP-2 titer determination, homogenized samples and lysed samples were used. For better comparability, the samples taken after E-lysis were solved in the homogenization buffer. One milliliter of each of these samples was centrifuged (16,600×*g*, 10 min, 4 °C), the supernatant was discarded, and the pellet was resuspended in the solubilization buffer (6 M GuanidinHCl, 50 mM β-mercaptoethanol, 10 mM Tris, 10 mM iodacetamide, pH 7.6) to a final protein concentration of 5 ± 1 g/l. For solubilization, samples were shaken overnight in 15-ml plastic tubes (Greiner Bio-One International AG, Kremsmünster, Austria).

Then, samples were transferred to a 1.5-ml reaction tube (Eppendorf, Hamburg, Germany) and centrifuged (20,815 RZB, 30 min). The supernatant was analyzed via HPLC (UltiMate 3000; Thermo Fisher, Waltham, MA, USA) with a reversed phase column (RP-1S, Thermo Fisher, Waltham, MA, USA) using a gradient method (flow rate 0.5 ml/min; eluent 95% acetonitrile, 5% H_2_O, 0.1% trifluoric acid; linear gradient 0–100% eluent in 10 min, 30 °C, detection at 280 nm).

#### Flow cytometry: investigation of E-lysis efficiency

To detect the advance in protein E-mediated lysis, flow cytometry was conducted at-line for each sample after the start of the E-lysis phase in all cultivations. To do so, a Cube 6 system (Partec, Münster, Germany) was used. The machine had a 488-nm blue solid state laser, whereas, five optical parameters were available. These were three fluorescence channels as well as a forward scatter (FSC) and side scatters (SSC). The wavelengths detected by the fluorescence channels (FL) were 527 nm for FL1 (band-pass filter, bandwidth 30 nm), 590 nm for FL2 (band-pass filter, bandwidth 50 nm), and 630 nm for FL3 (long-pass filter).

A combination of fluorescence stains RH 414 (red, abs./em.: 532/718 nm) and DiBAC4(3) (green, abs./em.: 493/516 nm) (both: AnaSpec, Fremont, CA, USA) as well as FSC were used to evaluate the E-lysis efficiency which was defined as the percentage of BGs in the total cell population. Sample preparation, flow cytometry measurement, and data treatment were conducted as described by Langemann et al. (2016).

## Results

### General process description

**Fig. 2:**
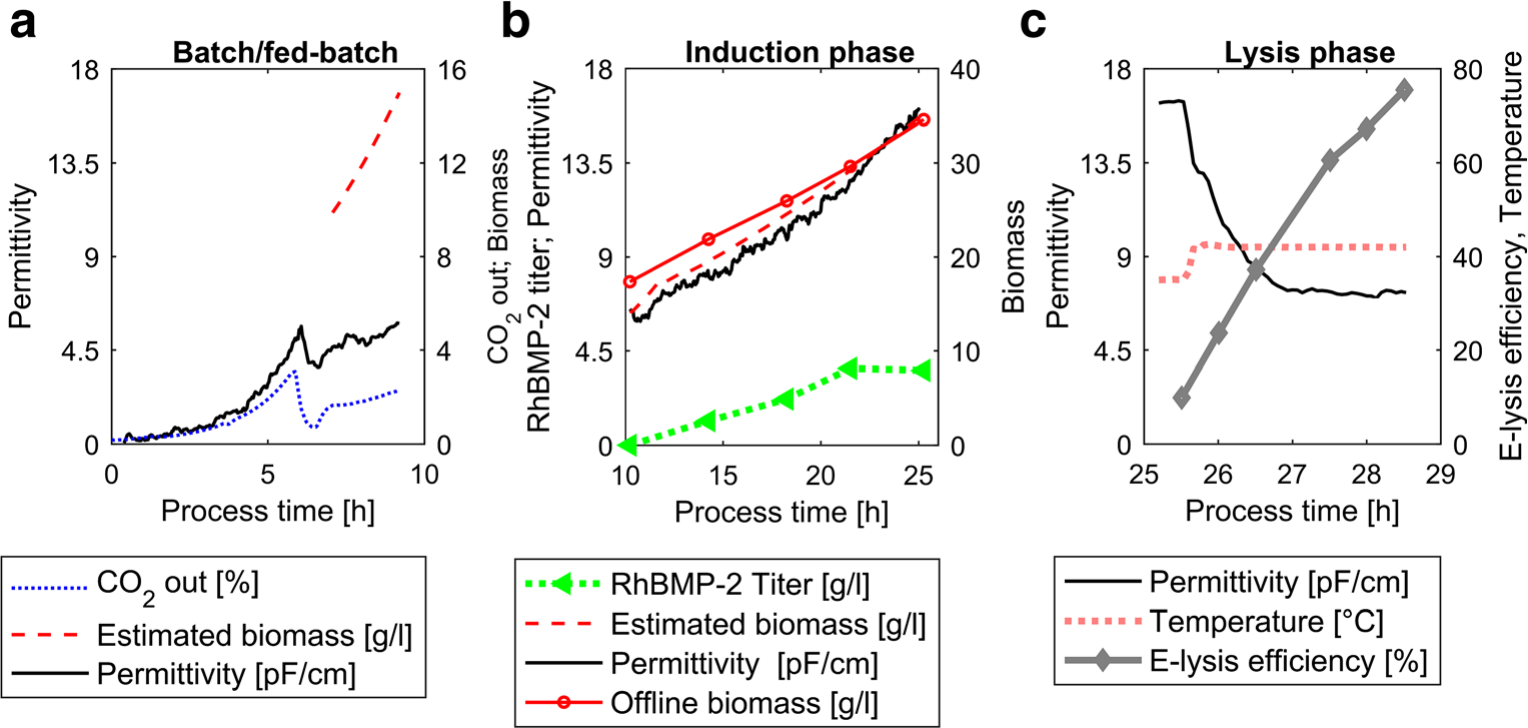
Online measurements of one cultivation (FB5). **a** Batch and fed-batch phase. Permittivity signal and CO_2_ in the off-gas are shown. Additionally, the biomass estimated by the soft-sensor in the fed-batch phase can be seen. **b** Induction phase. Estimated biomass is compared to offline biomass measurements. Furthermore, the Permittivity signal and the volumetric product titer are shown. **c** E-lysis phase. The E-lysis phase begins with the temperature shift. E-lysis efficiency and the Permittivity measurements are shown as well

To better understand the four different phases of the cultivations presented here as well as the mentioned process control strategies, one point of the DoE (FB5) is now going to be described in more detail. This cultivation is represented divided into process phases in Fig. 2: the batch and fed-batch (for biomass growth), the induction phase with a mixed feed, and the E-lysis phase.

During the batch phase, biomass accumulated to a concentration of approximately 10 g/l. This increase of biomass concentration is reflected in the CO_2_ off-gas signal and in the Permittivity signal (Fig. 2a). After a decrease in the CO_2_ off-gas signal, indicating the end of batch phase, a feed-forward-controlled fed-batch with D-glucose as sole carbon source was started. Subsequently, the soft-sensor for the estimation of the biomass concentration was started (Fig. 2a). The initial biomass concentration was, for this purpose, estimated via OD correlation and subsequently used as a starting value. As soon as the soft-sensor estimated a biomass of 15 g/l, the D-glucose feed was stopped and a pulse of L-arabinose to a final concentration of 2.5 g/l was performed. Following the depletion of the L-arabinose pulse, as monitored via a decrease in the CO_2_ off-gas signal (not shown), a second feed (mixture of D-glucose and L-arabinose) was started to begin the mixed feed induction phase.

The soft-sensor-assisted control strategy was used to control the desired set points of specific D-glucose and L-arabinose uptake according to the DoE set points. Figure 2b shows the accordance of estimated biomass based on the soft-sensor and the offline determined DCW. Following the formation of 35 g/l biomass as estimated according to the soft-sensor, the L-arabinose/D-glucose mixed feed was stopped. The feed was changed to D-glucose in order to run a specific substrate uptake rate of D-glucose at a set point of 0.8 g/(g × h). Due to lysis, the soft-sensor is not applicable in the E-lysis phase. Hence, the Permittivity signal was applied for the control of *q*_s_ D-glucose, using pre-established calibration to estimate biomass concentration during induction phase. Subsequently, the reactor temperature was shifted from 35 to 42 °C to induce the expression of the recombinant gene E, thereby starting the E-lysis phase (Fig. 2c). The Permittivity signal after the start of the E-lysis phase was found to decrease sharply in a negative exponential way, aligning well with the increase of E-lysis efficiency, as measured via flow cytometry measurements.

### Investigating the impact of the specific growth rate and L-arabinose feed fraction on the E-lysis efficiency

E-lysis efficiency is defined as the percentage of lysed cells in respect to the total cell population. In this study, achieved maximal E-lysis efficiencies varied between 31% in FB10 and 99% in FB3. Evaluation using multilinear regression analysis showed that the specific growth rate and the concentration of L-arabinose in the feed significantly impact the E-lysis efficiency (*R* ^2^: 0.83). The model for these linear effects had an *F* value of 14.4 (significant *F* value: 0.005). Both trends were significant with a *p* <0.05.

**Fig. 3:**
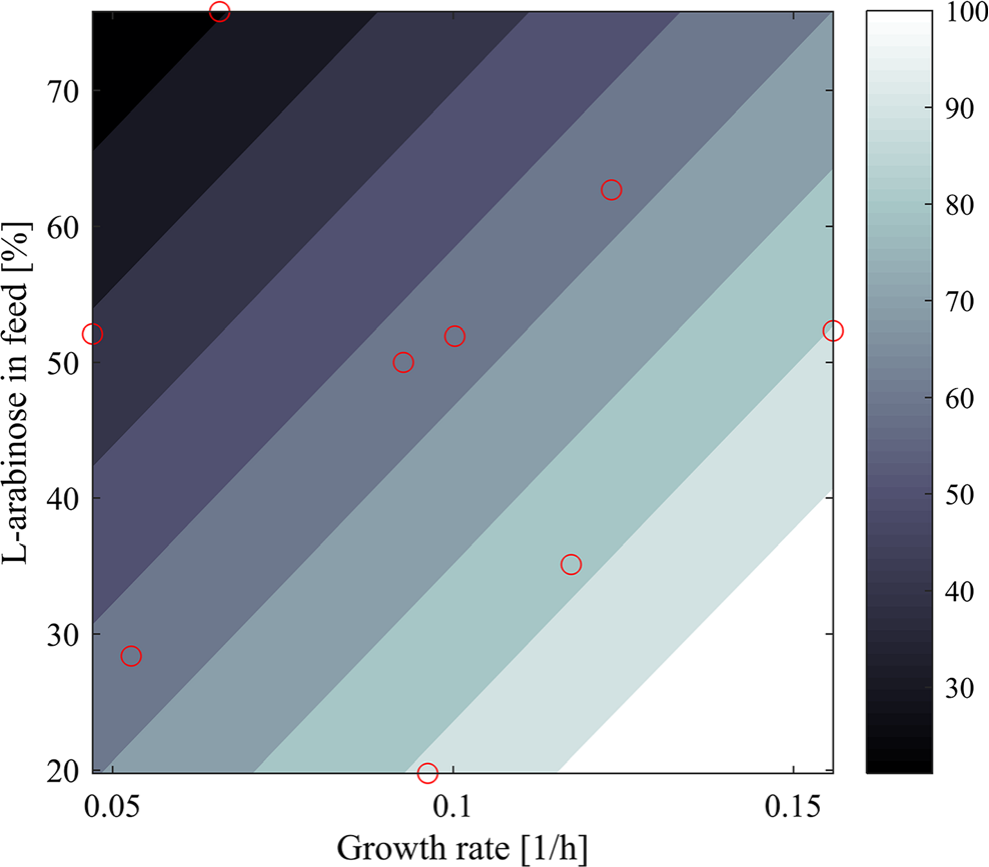
Prediction plot for E-lysis efficiency as a function of L-arabinose in the feed and specific growth rate. The *red circles* indicate the experimental data

The impact of L-arabinose in the feed and the specific growth rate on the E-lysis efficiency is displayed in Fig. 3.

### Protein E-mediated lysis kinetics

Knowledge on E-lysis kinetics is important to assess the time needed for full lysis in the manufacturing process. In the investigated process, E-lysis efficiencies close to 99% were only achieved at high specific growth rates and low inducer uptake rates. This was concluded to be due to the formation of subpopulations not capable of performing protein E-mediated lysis.

The following analysis of the kinetics of protein E-mediated lysis is based on the cellular subpopulation that was found capable to undergo protein E-mediated lysis, in the following referred to as “E-lysis competent cell population.” This population was calculated on the basis of measured biomass dry cell weight concentrations and E-lysis efficiencies (see Eq. 2).

2 $$ \mathrm{E}-\mathrm{lysis}\ \mathrm{competent}\ \mathrm{cell}\ \mathrm{population}={c}_{x0}-{c_{x0}}^{\ast}\left(\frac{LE_t}{100}\right)-\left({c}_{x0}-{c_{x0}}^{\ast}\left(\frac{LE_{\mathrm{final}}}{100}\right)\right) $$

Equation 2: E-lysis competent cell population. The calculation is based on the E-lysis efficiency (LE) and the biomass concentration at the start of E-lysis (*c*_*x*0_).

**Fig. 4:**
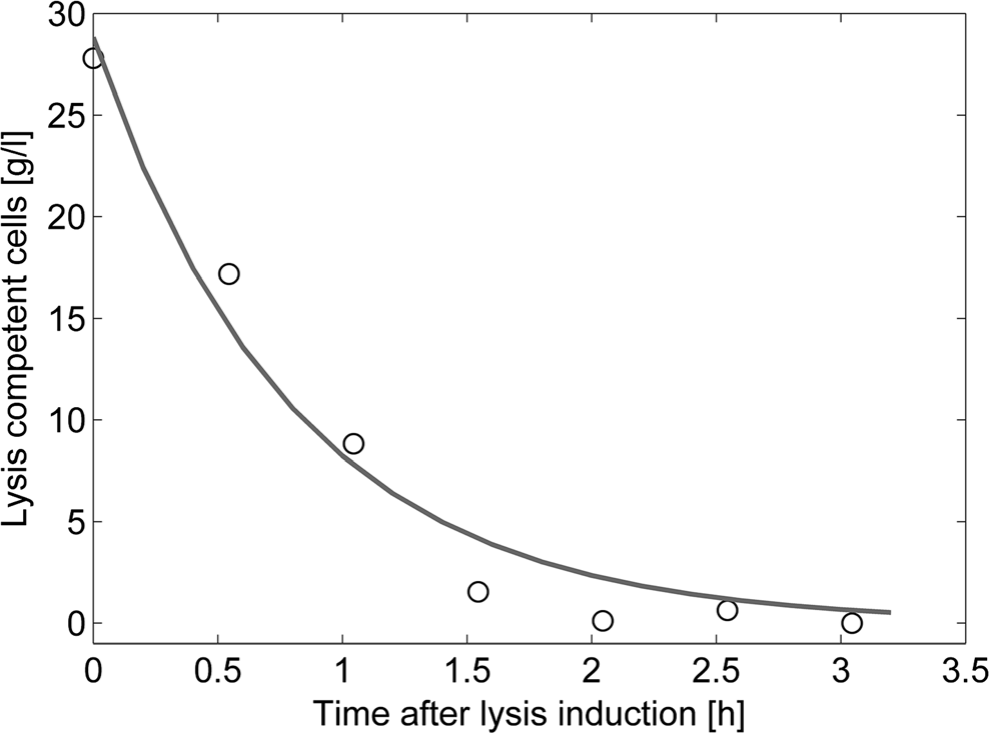
Trend of E-lysis competent cell population during E-lysis phase of FB3. The *circles* indicate the experimental data while the *black line* shows the trend

The number of E-lysis competent cells decreases with on-going E-lysis phase until it approaches 0 asymptotically at the end of this same phase. A negative exponential curve showed to appropriately fit the trend of E-lysis competent cells over time. Hence, the amount of E-lysis competent cells (*N*) over time (*t*) described as Eq. 3 shows *k* being the reaction constant. The function of E-lysis competent cells as a function of time was fitted for all process runs as exemplified in Fig. 4.

3 $$ \frac{aN}{dt_L}={k}^{\ast }{t}_L $$

Equation 3: First-order kinetics describing the trend of E-lysis competent cells over the E-lysis phase. The equation includes the number of E-lysis competent cells (*N*) over time of the E-lysis phase (*t*_L_) and the fitted reaction constant *k.*

The fitted reaction constant *k* was found to vary between −0.97 and −0.47, and, additionally, there was one outlier with a *k* of −1.25. This outlier belonged to FB3, which had the highest E-lysis efficiency of all fermentations (99%).

Multivariate investigation of interdependencies between the process and physiological parameters with E-lysis kinetics did not yield in any significant connections. Nevertheless, the mean reaction coefficient *k* was determined as −0.76 ± 0.29 and can be used to estimate the time necessary to reach maximal protein E-mediated lysis.

### Evaluation of product titer as a function of process parameters

**Fig. 5:**
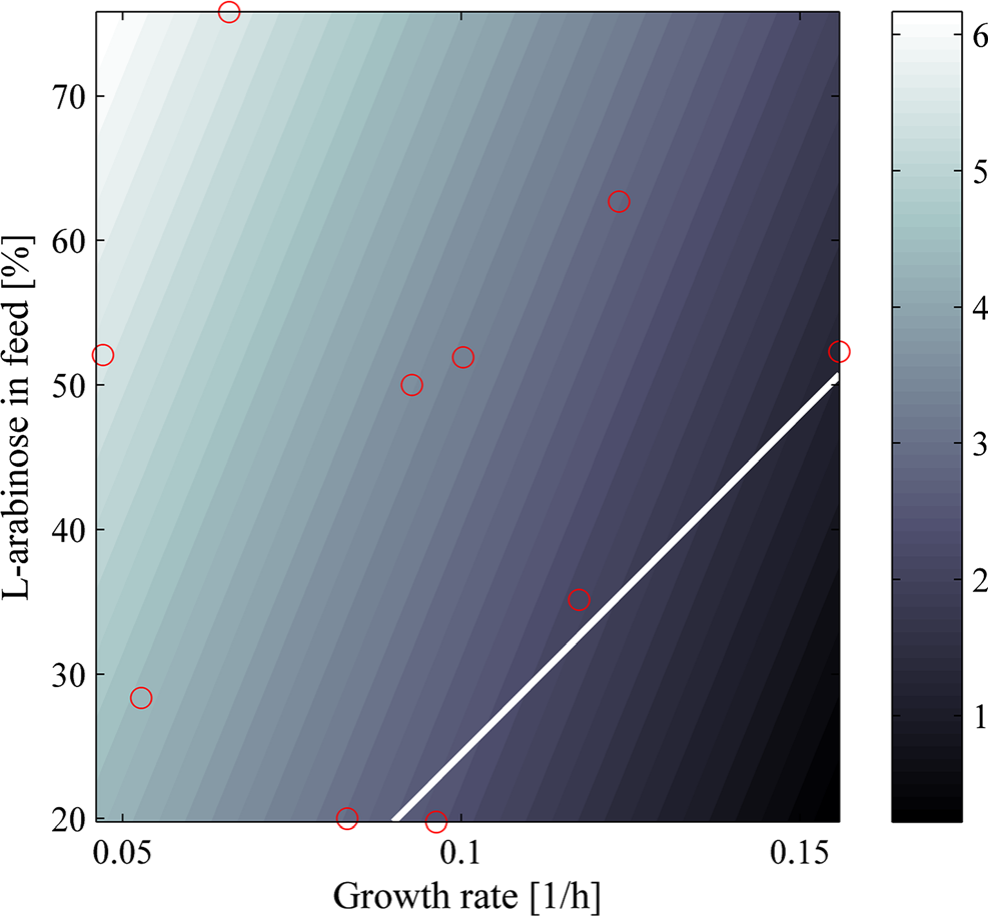
Prediction of product titer based on the linear regression model for different levels of growth rate and L-arabinose in the feed. The *white line* indicates the boundary below which 90% of E-lysis efficiency and more could be achieved. The *red circles* indicate the experimental data

As for the E-lysis efficiency, the influence of process parameters on the product titer at the end of the induction phase was investigated. The total specific substrate uptake rate showed a negative linear correlation to the final product titer, while L-arabinose in the feed was positively correlated. The model for these linear effects was significant with an *F* value 58.9 (significant *F* value: 4 × 10^−5^, *R* ^2^: 0.94). Both trends were significant with a *p* <0.05. Predictions of the product titer based on the model are shown in Fig. 5.

The conducted cultivations revealed a maximal product titer of 6.1 g/l at a growth rate of 0.05 h^−1^ (FB9). Maximal titers at E-lysis efficiencies higher than 90% were predicted to be 2.6 g/l at a growth rate of 0.09 h^−1^ and 19.8% L-arabinose in the feed.

For the upstream process, time-space-yields (gram rhBMP-2 per liter fermentation broth and process hour) were calculated for homogenized samples from the end of the induction phase and E-lysed samples. Yields were significantly higher for homogenized samples from the end of induction phase (0.74 ± 0.25 g/(l × h)) than E-lysed samples (0.47 ± 0.17 g/(l × h)), as a paired *t* test showed (*p*: 0.0119, *α*-level of 0.05). These time-space-yields did not consider downstream steps like homogenization. Thus, the 3-h elongated upstream process, resulting from the addition of the E-lysis phase, led to this difference. This was underlined by the findings that the rhBMP-2 recovery in grams per liter after solubilization of the homogenized respective of the E-lysed sample measured via HPLC analysis were the same. A paired *t* test revealed missing significance and thus supported the hypothesis that there was no significant difference in rhBMP-2 titer (*p*: 0.41, *α*-level of 0.05).

### Evaluation of inclusion body purity as a function of process parameters and primary recovery method (E-lysis vs. high-pressure homogenization)

In order to compare protein E-mediated lysis to “classical” cell rupture by means of high-pressure homogenization, the inclusion body purity was compared for the respective last samples of the induction phase in combination with homogenization to the sample after E-lysis (without homogenization). Inclusion body purity refers to the ratio of recombinant protein to other proteins found via SDS gel analysis in pellets of homogenized samples and BGs. Pellets of the latter contained the cell envelope with sealed periplasmatic space and inclusion bodies, while pellets of homogenized samples were expected to contain—besides inclusion bodies—only cell debris.

**Fig. 6:**
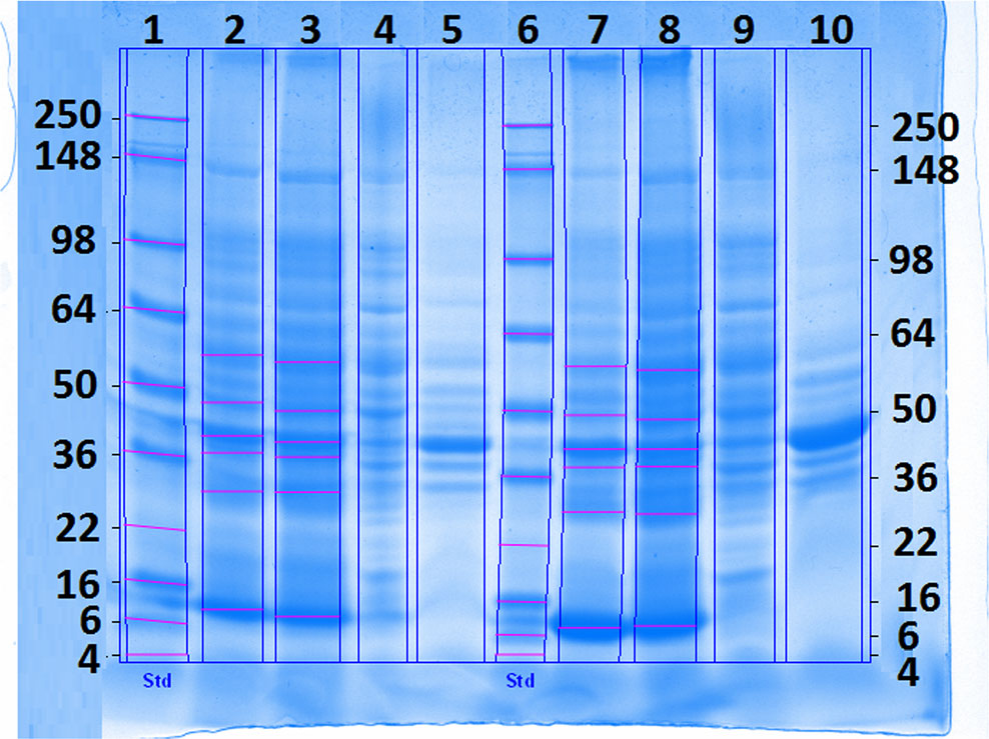
SDS gel for FB3 and FB5. *Lane 1* and *6*—standard; FB3: *lane 2*—last sample before lysis (homogenized pellet), *lane 3*—sample with highest E-lysis efficiency (pellet): *lane 4*—homogenization supernatant of the last sample before lysis, *lane 5*—fermentation supernatant of the last sample before lysis. For FB5, samples were loaded in the same order on *lanes 7 to 10*. rhBMP-2 was expected at 13 kDa

SDS gel analysis was carried out for all homogenized samples at the end of the mixed feed process, as well as for non-homogenized samples taken after the protein E-mediated lysis, in triplicate each. Furthermore, the fermentation broth supernatant and the homogenization supernatant were checked for the presence of soluble product (rhBMP-2). A typical SDS gel is displayed in Fig. 6. No soluble rhBMP-2 was detected in the fermentation and the homogenization supernatant as indicated by the absence of a 13-kDa band.

The impact of process parameters and cell rupture methods on the inclusion body purity was investigated. SDS gel analysis was carried out in triplicate for all homogenized end mixed feed phase samples and E-lysis phase samples for all processes. Densiometric evaluation showed that homogenized samples are significantly purer (61 ± 10%) than lysed non-homogenized samples (52 ± 7%), which was verified with a paired *t* test (*p* = 0.0015; *α* = 0.05). However, protein E-mediated ruptured samples were also found to be 40% purer compared to non-lysed and non-homogenized samples.

In high-pressure homogenization, all host cell proteins are expelled. Hence, only inclusion bodies and cell debris are found in the pellet used for SDS gel analysis. Opposed to this, solely cytoplasmic proteins are expelled during protein E-mediated lysis and the periplasmic space stays intact (Lubitz et al. 1999). The observation of higher amounts of host cell proteins in protein E-mediated samples could, hence, be due to retaining periplasmic host cell proteins in the BG. These are solubilized in the Laemmli buffer and hence loaded on the SDS gel.

The evaluation of a regression study identified a significant impact of the specific growth rate during the induction phase (*p* < 0.05) on the inclusion body purity after homogenization (*F* value 3.6, significant *F* value: 0.09, *R* ^2^: 0.50) as well as E-lysis (*F* value 6.5, significant *F* value: 0.03, *R* ^2^: 0.68) while the feed concentration of the inducer L-arabinose was found to be insignificant. At lower specific growth rates, inclusion bodies were found to be purer. At a specific growth rate of 0.05 h^−1^ (FB9), an inclusion body purity of 71 ± 4% in reference to key contaminating proteins was achieved after homogenization.

### Electron-microscopic investigation of inclusion body location after protein E-mediated cell lysis

**Fig. 7:**
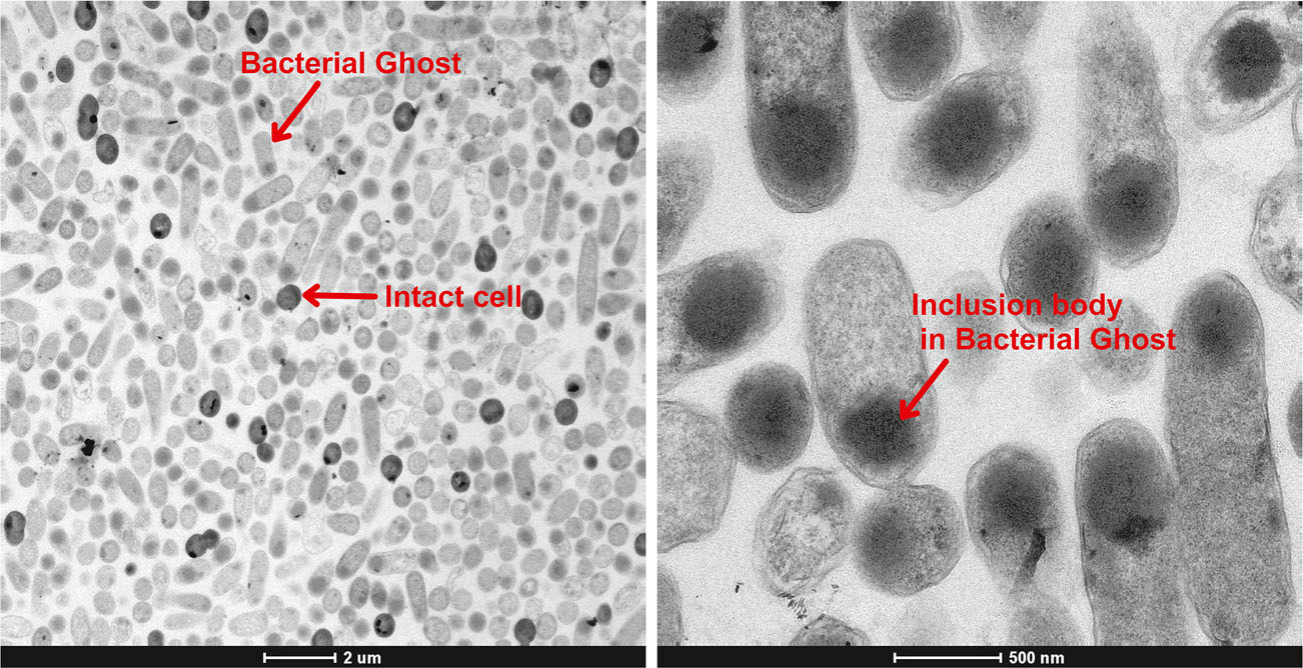
Microscope images of empty cell envelopes carrying the inclusion body. **a** Overview picture. **b** Picture with higher resolution

Figure 7a shows that BGs can clearly be distinguished from intact cells via electron microscopy. The comparison of cells indicated to be Bacterial Ghosts of respective intact cells reveals that. While intact cells are dense as a whole, BGs are translucent and partially include darker aggregates. The BGs being more translucent than intact cells was also the crucial criteria to distinguish BGs from intact cells in flow cytometry using the FCS. The ratio between the BGs and the intact cells on the microscopic pictures seems to accord with the flow cytometry measurements of the same sample. The phenomenon that BGs can be distinguished from intact bacteria by their higher transparence in the microscope was previously shown in a contribution as well using *E. coli* C41 containing the plasmids pBAD and pGLysivb (Kassmannhuber et al. 2017). Further, differences in transparence are deducible from the electron microscope pictures of Witte et al. (1998); therefore, we decided not to generate any further proof of this known phenomenon.

The principal message of these investigations with the electron microscope of samples after E-lysis phase was that inclusion bodies are found in the empty cell envelope (see Fig. 7b). This seems conceivable since border values of the transmembrane tunnel structure built by protein E were reported to be between 40 and 200 nm (Witte et al. 1992) whereas the size of inclusion bodies is typically in the range of 0.1 and 1.5 μm (Baneyx and Mujacic 2004). The comparison of the pictures of Fig. 7 to microscopic pictures of inclusion bodies in literature underlines these findings (Sriubolmas et al. 1997; Rinas and Bailey 1993).

## Discussion

### Metabolic load, pBAD, and E-lysis

The findings of E-lysis efficiencies below 90% at the end of fermentation can be explained by the formation of a subpopulation of viable but non-dividing cells due to the stress the cells undergo during recombinant protein production (Glick 1995). As expected, the amount of L-arabinose can be considered in close connection to the total recombinant gene expression and thus to a high stress/metabolic load. The metabolic load may alter their physiology of cells in a way to decrease their ability to E-lyse, as for example by alterations in growth (Hoffmann and Rinas 2004). In addition, high aggregation of intracellular proteins was reported to affect host proteins concerning oxidative stress and lipid metabolism, to affect the rearrangement of membrane lipid composition as well as to decrease the permeability and the fluidity of the membrane (Ami et al. 2009) — which again could be connected to the ability to E-lyse.

Furthermore, there is the influence of the specific growth rate. A possible explanation for this influence is the connection of the specific growth rate to the induction duration. The higher the growth rate, the faster a biomass concentration of 35 g/l is achieved. Longer induction time equals a longer period of stress exposure for the cell. In addition to that, the cell segregation during energy limited growth (Andersson et al. 1996) and recombinant protein production (Sundstrom et al. 2004) possibly has an impact on E-lysis efficiency. Thus, the cell energy is limited at too low growth rates which is coherent with cell segregation into viable but non-dividing cells (Andersson et al. 1996). Such cells, which are in an impaired physiological state that inhibits their division (Witte et al. 1987), may neither be able to E-lyse. This is supported by the dependence of the ability to E-lyse on the physiological state of the cell and its ability to divide as reported by Blasi et al. (1985) and Lubitz et al. (1984).

The pBAD mixed feed expression system was successfully used to tune recombinant protein production and to thereby control recombinant protein production to the extent of keeping the cells in an E-lysis-competent state (low L-arabinose in the feed). This highlights the power and versatility of this technology and suggests the application of the pBAD mixed feed system for other processes where the metabolic load on the cell factory is to be reduced by simple process technological means.

### E-mediated lysis as alternative to state-of-the-art methods

As stated above, state-of-the-art rupture methods to release recombinant protein (soluble or in the form of inclusion body aggregates) from the cytoplasm are detergent treatment, cell rupture by lysozyme, sonication, and freeze-thaw (Bird et al. 2004), as well high-pressure homogenization, bead milling, and thermolysis (Ren et al. 2007). These methods require at least one step in downstream processing, while the method developed here spares this downstream step, by adding one to upstream processing.

Besides the technology presented here, there exists another biological alternative to time and cost-intensive physical cell rupture. This is the excretion of recombinant proteins in the surrounding medium during the upstream process (Choi and Lee 2004; Sommer et al. 2010; Sommer et al. 2009). To do so, the target protein is fused with an N-terminal signal peptide enabling its transport to the periplasm. The coexpression of bacteriocin releases proteins, which degrade and permeabilize the outer membrane, and is reported to allow the diffusion of the target product from the periplasmic into the extracellular space (Sommer et al. 2009, 2010). However, this method requires soluble proteins which are transferable through the inner membrane: prerequisite for the release from the periplasmatic space, which is why the method is not applicable for the isolation of recombinant proteins produced as inclusion bodies. So far, inclusion body downstream processes demand cell rupture methods for the removal of soluble host cell proteins to isolate the target product in a highly concentrated and purified state (Fahnert et al. 2004).

This contribution showed that protein E-mediated lysis is an efficient means for the in-process rupture of cells to obtain recombinant protein from inclusion body purified from most host cell proteins. Using this technology, the downstream process can be facilitated by omitting high-pressure homogenization and adjacent steps, for example, clean in place (CIP) and steam in place (SIP) of the homogenizer. However, a significant quality loss in inclusion body purity (10%), due to retaining periplasmic proteins, as well as lower product titer, due to physiological constraints (specific growth rate >0.09 h^−1^, arabinose concentration in the feed <50%), is to be considered. An improvement of the solubilization procedure of the inclusion body in the empty cell envelope may increase product purity by omitting to open the periplasmic space. Thereby, the decrease of inclusion body purity by periplasmatic protein extraction could be omitted. An option could be the usage of milder detergents for solubilization. Applying non-denaturating detergents for solubilization enables gaining active protein from inclusion bodies without further renaturation steps (Peternel et al. 2008).

Nevertheless, the application of the presented technology for recombinant protein production in inclusion body processes has the potential of saving resources in downstream processing of pharmaceutical plants. Whether the costs could be decreased using this method must be evaluated case by case.

Additionally, this method offers the possibility to utilize Bacterial Ghosts including an inclusion body containing active proteins as a desirable product. In contrast to the widespread opinion that inclusion body proteins need to be solubilized to catalyze reactions, it was shown that enzymes in inclusion bodies show native-like structures and enzymatic activity (García-Fruitós et al. 2005, 2007; Tokatlidis et al. 1991). Hence, empty cell envelopes containing functional inclusion bodies may be used to catalyze enzymatic reactions utilizing a protein in its cytoplasmatic space as active enzyme. This application enables the possibility to produce high amounts of enzymes without subsequent cell disruption and solubilization of aggregated recombinant proteins. A similar application was shown by Sührer et al. (2015) using native proteins immobilized by membrane anchors.

Summing up, the investigation presented here describes a tunable expression system capable of controlling the fitness of the cells based on tuning the recombinant protein production. The possibility of conducting E-lysis as a form of cell rupture is additionally presented: a method which could be able to spare one step in the downstream process. The information in this contribution includes E-lysis kinetics, physiological constraints, product titer, and product purity. The study provides all information necessary for the science-based design of inclusion body processes, using the phage *Φ*X174-derived lysis protein E in combination with the pBAD mixed feed platform.

## Electronic supplementary material

ESM 1 (PDF 127 kb)
