# Supplementary material for: A novel method to recover inclusion body protein from recombinant E. coli fed-batch processes based on phage ΦX174-derived lysis protein E
Source: Appl Microbiol Biotechnol. 2017 Apr 20;101(14):5603–14. doi: 10.1007/s00253-017-8281-x (PMC5501905; doi:10.1007/s00253-017-8281-x)
Supplement: Supplementary file 1 — (PDF 127 kb) [file 253_2017_8281_MOESM1_ESM.pdf]

Applied Microbiology and Biotechnology

A novel method to recover inclusion body protein from recombinant *E. coli* fed-batch processes based on phage ΦX174 derived lysis protein E

Daniela Ehgartner[1,2,a], Patrick Sagmeister[1,5,a], Timo Langemann[3,4], Andrea Meitz[3], Werner Lubitz[4] and Christoph Herwig*[,1,2]

[a] these two authors contributed to this work equally

***to whom the correspondence should be addressed to**

[1] Institute of Biochemical Engineering, Vienna University of Technology, Vienna, Austria

[2] CD Laboratory on Mechanistic and Physiological Methods for Improved Bioprocesses, Vienna University of Technology, Austria

[3] Research Center of Pharmaceutical Engineering (RCPE) GmbH, Graz, Austria

[4] BIRD-C GmbH, Vienna, Austria

[5] Exputec GmbH, Vienna, Austria

Corresponding author:

christoph.herwig@tuwien.ac.at

Tel (Office): +43 1 58801 166400
Fax: +43 1 58801 166980

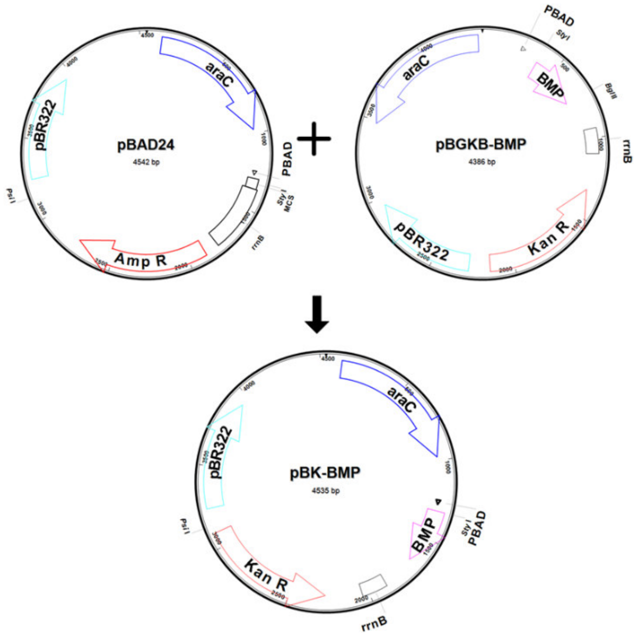

Fig. S1 The plasmid pBK-BMP
